# The Impact of Antenatal Lactation Counseling on Breastfeeding Practices and Newborn Feeding Behavior Among Primipara Mothers Attending a Tertiary Care Hospital in Puducherry, India: A Randomized Controlled Trial

**DOI:** 10.7759/cureus.113233

**Published:** 2026-07-23

**Authors:** Barsharani Behera, Kanimozhi K, Adhisivam Bethou

**Affiliations:** 1 Obstetrics and Gynaecological Nursing, College of Nursing, Jawaharlal Institute of Postgraduate Medical Education and Research, Puducherry, IND; 2 Obstetrics and Gynaecology, Kalinga Institute of Nursing Sciences, Kalinga Institute of Industrial Technology (Deemed to be University), Bhubaneswar, IND; 3 Neonatology, Jawaharlal Institute of Postgraduate Medical Education and Research, Puducherry, IND

**Keywords:** breastfeeding practices, feeding behaviour., lactation counselling, newborn, s: antenatal

## Abstract

Introduction

Although human milk provides unparalleled immunobiological and nutritional advantages essential for optimal neonatal development, the establishment of successful lactogenesis and sustained breastfeeding remains a complex psychophysiological challenge, particularly for primiparous mothers. First-time parturients frequently encounter biomechanical difficulties, such as suboptimal infant positioning and latch, which are compounded by postnatal anxiety. These factors can disrupt oxytocin-mediated milk let-down and precipitate disorganized neurobehavioral feeding reflexes in the newborn. Traditional postnatal lactation support is often reactive, administered during a period of high maternal fatigue and physiological stress. Therefore, structured antenatal lactation counseling emerges as a critical, proactive intervention designed to enhance maternal self-efficacy, provide anticipatory guidance, and mitigate the early introduction of prelacteal feeds. This randomized controlled trial (RCT) aimed to empirically validate this paradigm shift within a localized, high-volume clinical setting. It sought to investigate the effectiveness of targeted antenatal lactation counseling on both maternal breastfeeding practices and newborn feeding behaviors among primiparous mothers attending a tertiary care hospital in Puducherry, ultimately seeking to optimize perinatal care protocols through rigorous, evidence-based intervention.

Methods

An RCT was conducted with 130 primiparous women aged 18 to 35 years who had completed 32 weeks of gestation and delivered at term. A group counseling technique was used for the intervention group, wherein groups of five mothers received 30-minute antenatal lactation counseling sessions during antenatal visits. The lactation counseling on breastfeeding was repeated three times at 32, 34, and 36 weeks of gestation, while the control group received only routine care. Breastfeeding practices and newborn feeding behavior were assessed using a structured breastfeeding practices checklist and the Infant Breastfeeding Assessment Tool (IBFAT) on the second postnatal day.

Results

Baseline variables did not differ significantly between the groups. The intervention group showed significantly better breastfeeding practices (t = 5.384, p = 0.000) and newborn feeding behavior (t = 3.22, p = 0.02). Breast engorgement was lower in the intervention group, though the difference was not statistically significant.

Conclusions

Based on our findings, proper lactation counseling provided to primiparous mothers at each contact point during antenatal visits improves breastfeeding practices and promotes optimal newborn feeding behavior.

## Introduction

Newborns have the best chance of surviving, developing, and realizing their full potential when their mothers breastfeed them because human breast milk has unique biological properties and a dynamic, interactive composition [1]. In India, over 1.1 million infants die within their first month of life, with an additional 500,000 dying between two and 12 months of age [2]. Inadequate breastfeeding contributes to approximately 16% of child deaths annually [3]. Early breastfeeding cessation is frequently caused by a mother's lack of confidence in her ability to breastfeed, problems with the infant latching or suckling, breast pain or soreness, perceived insufficient milk supply, and a lack of individualized support from clinicians in the early post-discharge phase.

Addressing these issues requires antenatal education on the benefits of breastfeeding and mental preparation for exclusive breastfeeding [4]. Antenatal education is essential when the maternity stay after delivery is shorter, as there is limited time to learn about breastfeeding after birth. Prenatal visits allow health professionals to assess women’s breastfeeding knowledge and provide guidance on effective breastfeeding management [5]. In southern India, the impact of antenatal lactation counseling has been the subject of limited published research. Hence, this study was conducted to determine the effect of antenatal lactation counseling on postpartum mothers’ breastfeeding practices.

## Materials and methods

Study design and setting

This randomized controlled trial (RCT) was conducted at a tertiary care teaching institute in South India from October 2023 to January 2024 after obtaining ethical approval from the Institutional Ethics Committee (IEC No. JIP/CON/IEC/M.Sc./2022/OBG/1) and was prospectively registered with the Clinical Trials Registry of India (CTRI/2023/09/057815).

Study population and sample

This study included primiparous mothers aged 18-35 years who attended regular antenatal check-ups and planned to deliver at the same institution. Mothers whose babies were admitted to the neonatal intensive care unit (NICU) were excluded from the study. The sample size was calculated by considering the proportion of good breastfeeding practices in the control group as 50%, based on the reference article [6], and assuming that the proportion of good breastfeeding practices would be 25% higher in the intervention group. Using a 5% level of significance, 80% power, and accounting for a 10% attrition rate, a total of 130 mothers were enrolled in the study, with 65 participants allocated to each group.

Randomization and intervention

After obtaining informed consent, these mothers were enrolled in the study at 32 weeks of gestation and randomized into either the intervention or control group. A computer-generated randomization sequence was used, and allocation concealment was achieved through the serially numbered, opaque, sealed envelope (SNOSE) technique. After obtaining baseline data, a group counseling technique was used for the intervention group, wherein groups of five mothers were counseled for 30 minutes during antenatal visits. The lactation counseling on breastfeeding was repeated three times at different intervals, i.e., at 32 weeks, 34 weeks, and 36 weeks of gestation, while the control group received only routine care. Mothers in the intervention group were counseled regarding the meaning of exclusive breastfeeding, the timing of breastfeeding initiation, type and composition of breast milk, the importance of breastfeeding, breastfeeding techniques including positioning, frequency and adequacy of breastfeeding, newborn feeding behavior, and the prevention and management of breast engorgement.

Data collection and assessment

For outcome assessment, the mothers were assessed on the second postnatal day after delivery using a structured, faculty-validated, pretested checklist to evaluate breastfeeding practices. The checklist consisted of 17 questions with two response options. A correct answer was scored as 1, and an incorrect answer was scored as 0. The maximum possible score was 17, and the minimum score was 0. The level of breastfeeding practices was interpreted as good practice (13-17), fair practice (8-12), and poor practice (0-7). Newborn feeding behavior was assessed using the Infant Breastfeeding Assessment Tool (IBFAT) [7], a standardized tool. On the third postnatal day, the occurrence of breast engorgement was assessed through a telephone conversation to determine whether breast engorgement was present.

The content validity of the tool was assessed using the item-level content validity index (I-CVI/Avg) for the structured breastfeeding practices checklist. The obtained validity score was 0.97, indicating that the tool was valid, as an I-CVI/Avg value ranging from 0.9 to 1 is considered acceptable. The validity of the tool was determined based on its relevance, sufficiency, and appropriateness. The Cronbach’s alpha method was used to evaluate the reliability of the tool, and the obtained reliability coefficient was 0.759.

Statistical analysis

The data were tabulated and summarized in Microsoft Excel sheets and analyzed and interpreted using descriptive and inferential statistics. A p-value of <0.05 was considered statistically significant.

**Figure 1:**
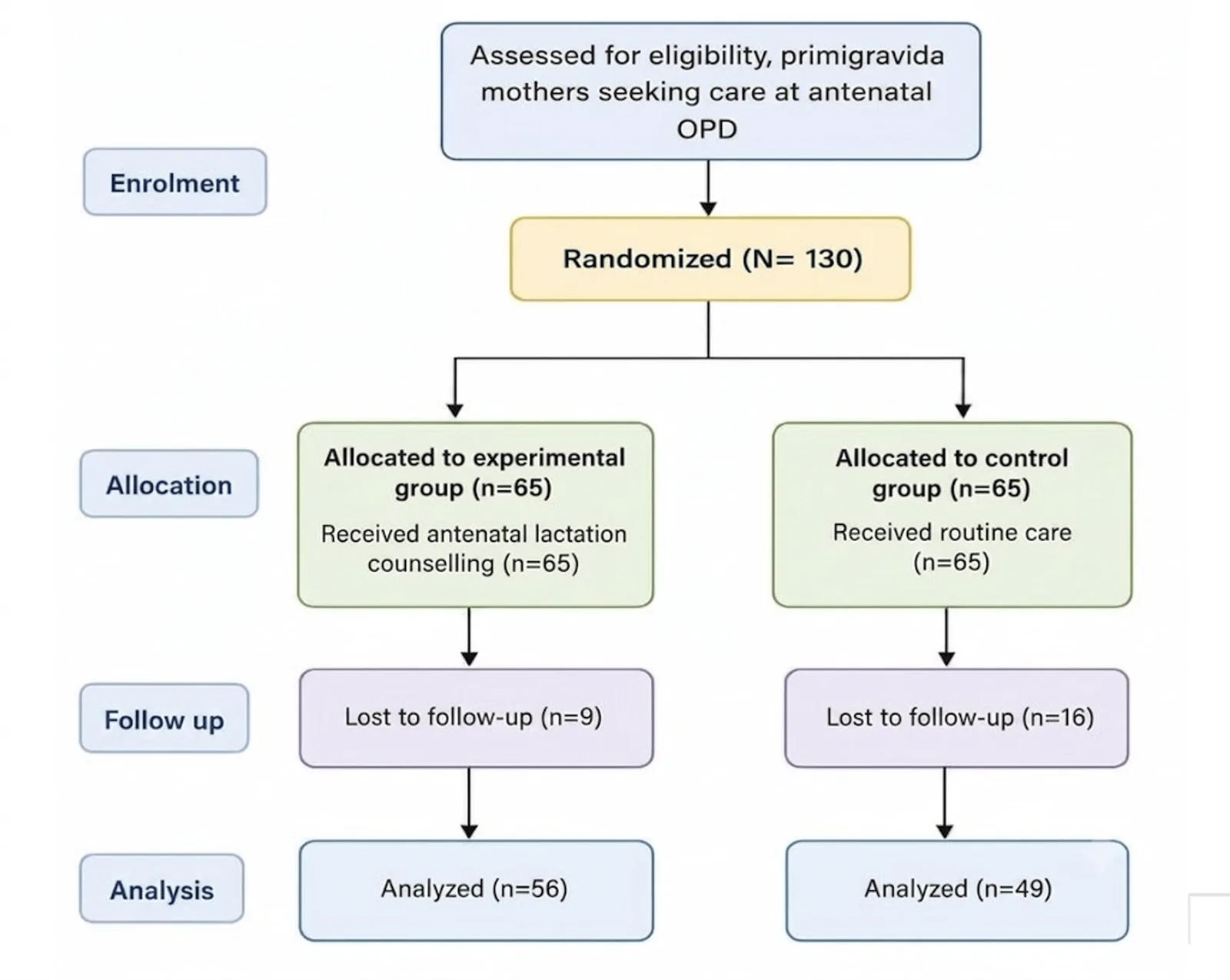
CONSORT flow diagram depicting the flow of participants CONSORT: Consolidated Standards of Reporting Trials; OPD: outpatient department

Figure 1 illustrates the flow of participants through the RCT. A total of 130 primigravida mothers attending the antenatal outpatient department (OPD) were assessed for eligibility and subsequently randomized into two equal groups (n = 65 each). The experimental group received antenatal lactation counseling, while the control group received routine antenatal care. During the follow-up period, nine participants from the experimental group and 16 participants from the control group were lost to follow-up. Consequently, 56 participants in the control group and 49 participants in the experimental group were included in the final analysis. The flowchart demonstrates the process of enrolment, randomization, allocation, follow-up, and analysis of study participants, ensuring transparency in participant progression throughout the study.

## Results

A total of 130 primipara mothers were randomized to the study. After attrition during follow-up, 105 mothers completed the study and were included in the final analysis, comprising 56 mothers in the intervention group and 49 mothers in the control group. The baseline demographic and clinical characteristics of the participants are presented in Table 1. The majority of mothers in both groups were aged 24-29 years (55.4% vs. 53.1%). None of the participants lacked formal education, and most had completed a diploma or higher qualification (60.7% vs. 69.4%). Homemakers constituted the majority of participants in both groups (87.5% vs. 91.8%). Approximately half of the participants had been married for one to three years, belonged to joint families, and reported a monthly family income below ₹10,000. Around three-quarters had not previously received breastfeeding information. Among those who had, healthcare professionals were the most common source. Overall, the two groups were comparable at baseline (Table 1).

**Table 1:** Baseline variables in intervention and control groups (N = 105)

Sl. No	Demographic and clinical variables	Intervention (n = 56), n (%)	Control (n = 49), n (%)
1	Age group, years		
	18-23	18 (32.1)	13 (26.5)
	24-29	31 (55.4)	26 (53.1)
	30-35	7 (12.5)	10 (20.4)
2	Education		
	No formal education	0 (0.0)	0 (0.0)
	Up to higher secondary	22 (39.3)	15 (30.6)
	Diploma and above	34 (60.7)	34 (69.4)
3	Occupation		
	Homemakers	49 (87.5)	45 (91.8)
	Employed	7 (12.5)	4 (8.2)
4	Duration of marriage, years		
	< 1	13 (23.2)	9 (18.4)
	1-3	28 (50.0)	28 (57.1)
	3-5	10 (17.9)	7 (14.3)
	> 5	5 (8.9)	5 (10.2)
5	Type of family		
	Joint	37 (66.1)	36 (73.5)
	Nuclear	19 (33.9)	13 (26.5)
6	Family monthly income, INR		
	< 10,000	32 (57.1)	34 (69.4)
	10,000-20,000	16 (28.6)	9 (18.4)
	> 20,000	8 (14.3)	6 (12.2)
7	Information obtained regarding breastfeeding practices previously		
	Yes	14 (25.0)	11 (22.4)
	No	42 (75.0)	38 (77.6)
8	Source of information		
	Family members	4 (7.8)	3 (6.1)
	Healthcare professionals	10 (19.6)	8 (16.3)
	Mass media	0 (0.0)	0 (0.0)

The breastfeeding practices of the mothers are presented in Table 2. Most primipara mothers, 78.6% in the intervention group and 73.5% in the control group, initiated breastfeeding within one hour. No participants discarded colostrum or gave prelacteal feeds in either group. In the intervention group, all the primipara mothers remained calm and relaxed while breastfeeding, took adequate water and food before breastfeeding, and fed their babies every two hours, whereas in the control group, almost all the primipara mothers (48, 98.0%) remained calm while breastfeeding, the majority of them (44, 89.8%) took adequate water and food before breastfeeding, and 46 (93.9%) fed their babies every two hours (Table 2).

**Table 2:** Breastfeeding practices in intervention and control groups (N = 105)

Sl. No.	Items	Intervention (n = 56), no, n (%)	Intervention (n = 56), yes, n (%)	Control (n = 49), no, n (%)	Control (n = 49), yes, n (%)
1	Did you initiate breastfeeding within one hour?	12 (21.4)	44 (78.6)	13 (26.5)	36 (73.5)
2	Did you give the colostrum to your baby?	0 (0)	56 (100.0)	0 (0)	49 (100.0)
3	Did you give your baby honey, plain water, sugar water, or other milk?	56 (100.0)	0 (0)	49 (100.0)	0 (0)
4	Do you remain calm and relaxed while breastfeeding your baby?	0 (0)	56 (100.0)	1 (2.0)	48 (98.0)
5	Did you take adequate water and food before breastfeeding?	0 (0)	56 (100.0)	5 (10.2)	44 (89.8)
6	Did you sit comfortably with your back straight while breastfeeding?	3 (5.4)	53 (94.6)	7 (14.3)	42 (85.7)
7	Do you keep the baby's head, neck, and body in the same plane?	3 (5.4)	53 (94.6)	6 (12.2)	43 (87.8)
8	Do you ensure the baby's mouth widens, and the majority of the areola is inside the baby's mouth while breastfeeding?	1 (1.8)	55 (98.2)	6 (12.2)	43 (87.8)
9	Do you allow the baby to feed on one breast till the baby stops sucking and releases the breast?	5 (8.9)	51 (91.1)	9 (18.4)	40 (81.6)
10	Do you burp the baby immediately after breastfeeding?	3 (5.4)	53 (94.6)	7 (14.3)	42 (85.7)
11	Do you offer the other breast for the next feed?	6 (10.7)	50 (89.3)	8 (16.3)	41 (83.7)
12	Do you clean your breasts each time before and after feeding the baby?	1 (1.8)	55 (98.2)	2 (4.1)	47 (95.9)
13	Do you breastfeed your baby every 2 hours?	0 (0)	56 (100.0)	3 (6.1)	46 (93.9)
14	Do you breastfeed your baby on demand during the day and night?	2 (3.6)	54 (96.4)	2 (4.1)	47 (95.9)
15	Is the baby able to establish an effective sucking pattern on both breasts (initial rapid sucks, then slower sucks with pauses)?	4 (7.1)	52 (92.9)	4 (8.2)	45 (91.8)
16	Do you use artificial pacifiers or teats for your baby?	56 (100.0)	0 (0)	49 (100.0)	0 (0)
17	Do you monitor the urine frequency and pattern of sleep after each feed to assess the adequacy of milk intake?	4 (7.1)	52 (92.9)	8 (16.3)	41 (83.7)

The overall breastfeeding practice scores are presented in Table 3. None of the mothers in either group demonstrated poor breastfeeding practices. Good breastfeeding practices were observed in 96.4% of the intervention group compared to 71.4% of the control group. The mean breastfeeding practice score was significantly higher in the intervention group than in the control group (16.17 ± 1.40 vs. 14.51 ± 1.76; t = 5.384, df = 103, p < 0.001), indicating that antenatal lactation counseling significantly improved breastfeeding practices among primipara mothers (Table 3).

**Table 3:** Level of breastfeeding practices in intervention and control groups (N = 105) *Statistically significant (p < 0.05) SD: standard deviation

Level of breastfeeding practice (score)	Intervention (n = 56), n (%)	Intervention (n = 56), mean ± SD	Control (n = 49), n (%)	Control (n = 49), mean ± SD	df	t-value	P-value
Poor practice (0-7)	0 (0)	16.17 ± 1.40	0 (0)	14.51 ± 1.76	103	5.384	0.000^*^
Fair practice (8-12)	2 (3.6)		9 (16.1)				
Good practice (13-17)	54 (96.4)		40 (71.4)				

The newborn feeding behavior scores are presented in Table 4. Vigorous and effective feeding was observed in 91.1% of newborns in the intervention group compared to 62.5% in the control group. Moderately effective feeding was observed in 8.9% and 25.0% of newborns, respectively, while no newborn demonstrated poor feeding behavior. The mean feeding behavior score was significantly higher in the intervention group (11.55 ± 1.67) than in the control group (11.44 ± 1.15) (t = 3.221, df = 103, p = 0.002), demonstrating the effectiveness of antenatal lactation counseling (Table 4).

**Table 4:** Newborn feeding behavior in intervention and control groups (N = 105) ^*^Statistically significant (p < 0.05) SD: standard deviation

Level of newborn feeding behavior (score)	Intervention (n = 56), n (%)	Intervention (n = 56), mean ± SD	Control (n = 49), n (%)	Control (n = 49), mean ± SD	df	t-value	P-value
Poor feeding (0-6)	0 (0)	11.55 ± 1.67	0 (0)	11.44 ± 1.15	103	3.221	0.002^*^
Moderately effective feeding (7-9)	5 (8.9)		14 (25)				
Vigorous effective feeding (10-12)	51 (91.1)		35 (62.5)				

The incidence of breast engorgement is presented in Table 5. In the intervention group, the majority of the primipara mothers (53, 94.6%) had no breast engorgement. Very few of them (3, 5.4%) had breast engorgement, whereas in the control group, 42 (75%) had no breast engorgement, and seven (12.5%) had breast engorgement; breast engorgement was less prevalent in the intervention group. However, the difference was not statistically significant (Table 5). The intervention group showed significant improvement in breastfeeding practices (t = 5.384, p = 0.00) and newborn feeding behavior (t = 3.22, p = 0.02), proving that antenatal lactation counseling effectively improves breastfeeding practices and newborn feeding behavior (Tables 3-4).

**Table 5:** Breast engorgement in intervention and control groups (N = 105) *Statistically significant (p < 0.05) SD: standard deviation

Presence of breast engorgement (score)	Intervention (n = 56), n (%)	Intervention (n = 56), mean ± SD	Control (n = 49), n (%)	Control (n = 49), mean ± SD	df	t-value	P-value
No	53 (94.6)	0.05 ± 0.22	42 (75)	0.14 ± 0.35	103	1.550	0.122^*^
Yes	3 (5.4)		7 (12.5)				

## Discussion

The present study showed that more than half of the primipara mothers in the intervention (31, 55.4%) and control (26, 53.1%) groups were aged between 24 and 29 years. None of the participants were illiterate in either the intervention or the control group. The majority of participants in the intervention group (49, 87.5%) and the control group (45, 91.8%) were homemakers. Half of the primipara mothers in the intervention group (28, 50.0%) and more than half of those in the control group (28, 57.1%) had a duration of marital life between one and three years.

Even though only a small percentage of participants had previously acquired information about breastfeeding practices in the intervention group (14, 25%) and the control group (11, 22.4%), the sources of information were family members (10, 19.6%) in the intervention group and eight (16.3%) in the control group, and healthcare professionals (10, 19.6%), respectively. None of the participants had received information from the media, although they were all literate (100%). This may be due to their ineffective use of the media. This highlights the importance of health professionals encouraging mothers to breastfeed and motivating them to use the mass media to obtain helpful information. A study conducted by Akadri and Odelola [8] also reported that women who received information on breastfeeding from the mass media were more likely than those who received information from other sources to breastfeed exclusively for six months.

In the present study, the majority of primipara mothers in the intervention group (78.6%) started breastfeeding within one hour. None of the mothers gave prelacteal feeds, and none discarded the colostrum. They all consumed enough food and water before breastfeeding, fed their infants every two hours, and stayed composed and at ease throughout the process. The study's results align with those of Chethana et al. [9], which found that 85% of mothers had good attachment, 84.1% burped their babies after feeding, 95.3% fed colostrum to their newborns, 25.2% had given prelacteal feeds, and 60.7% of mothers had started breastfeeding within one hour. On the other hand, the percentage of mothers who started breastfeeding within one hour was significantly greater than that reported by Bethou et al. [10], where 53.3% of mothers in the intervention group initiated breastfeeding within one hour of delivery.

According to the current study, none of the mothers in the intervention group had poor breastfeeding practices: 3.6% had fair breastfeeding practices, and 96.4% had good breastfeeding practices. This proportion was higher than that reported in a related study by Yadav et al. [4], in which the intervention group's mothers had good breastfeeding practices (63.4%), fair breastfeeding practices (33.3%), and poor breastfeeding practices (3.3%). Similar results were found in another study by Thomas et al. [11], which found that none of the mothers in the intervention group had poor breastfeeding practices at the post-test, 85% had good breastfeeding practices, and 14% had average breastfeeding practices.

In the present study, it was observed that the majority of newborns in the intervention group (91.1%) demonstrated vigorous and effective feeding, while 8.9% demonstrated moderately effective feeding, and none demonstrated poor feeding behavior. In the control group, 62.5% of newborns demonstrated vigorous and effective feeding, 25.0% demonstrated moderately effective feeding, and none demonstrated poor feeding behavior. In contrast, a study conducted by Devi et al. [12] showed that 47.5% of babies had an inadequate feeding pattern. Another study by Reena et al. [13] revealed that the experimental group's newborn feeding behavior significantly increased (p < 0.001) among the study group's primigravidae.

Only 5.4% of primipara mothers in the current study's intervention group reported breast engorgement, compared with 12.5% in the control group. The effectiveness of lactation counseling was demonstrated by a similar study conducted by Padmashree et al. [14], in which mothers in the experimental group reported 13.3% breast engorgement compared with 63.3% in the control group.

## Conclusions

The current study demonstrated the efficacy of antenatal lactation counseling in enhancing breastfeeding practices, improving newborn feeding behavior, and reducing breast engorgement. Therefore, as part of prenatal care, healthcare providers should offer antenatal lactation counseling to all primipara mothers. If proper counseling is provided during the prenatal period, breastfeeding practices can be strengthened, and breastfeeding difficulties can be addressed.

## Appendices

**Table 6:** Sociodemographic data

Sl. No.	Variable	Categories/options
1	Age (years)	a. 18–23 years; b. 24–29 years; c. 30–35 years
2	Education	a. No formal education; b. Up to higher secondary; c. Diploma and above
3	Occupation	a. Homemakers; b. Employed
4	Duration of marriage	a. < 1 year; b. 1–3 years; c. 3–5 years; d. > 5 years
5	Type of family	a. Joint; b. Nuclear
6	Family monthly income	a. < ₹10,000 b. ₹10,000–₹20,000 c. Above ₹20,000
7	Any information obtained regarding breastfeeding practices already?	a. Yes; b. No
8	If yes, mention the source of information	a. Family members; b. Healthcare professionals; c. Mass media

**Table 7:** Structured breastfeeding practices checklist This checklist consists of 17 questions. The right answer will be scored as 1, and the wrong answer will be scored as 0. The maximum possible score is 17, and the minimum score is 0. The level of breastfeeding practice based on the practice scores will be interpreted as good practice (13-17), fair practice (8-12), and poor practice (0-7)

Sl. No.	Items	Yes	No
1	Did you initiate breastfeeding within 1 h?		
2	Did you give the colostrum to your baby?		
3	Did you give honey, plain water, sugar water, or any other milk to your baby?		
4	Do you remain calm and relaxed while breastfeeding your baby?		
5	Did you take adequate water and food before breastfeeding?		
6	Did you sit comfortably with your back straight while breastfeeding?		
7	Do you keep the baby's head, neck, and body in the same plane?		
8	Do you ensure the baby's mouth widens, and the majority of the areola is inside the baby's mouth while breastfeeding?		
9	Do you allow the baby to feed on one breast till the baby stops sucking and releases the breast?		
10	Do you offer the other breast for the next feed?		
11	Do you burp the baby immediately after breastfeeding?		
12	Do you clean your breasts each time before and after feeding the baby?		
13	Do you breastfeed your baby every 2 h?		
14	Do you breastfeed your baby on demand during day and night?		
15	Is the baby able to establish an effective sucking pattern on both breasts (initial rapid sucks then slower sucks with pauses)?		
16	Do you use artificial pacifiers or teats for your baby?		
17	Do you monitor the urine frequency and pattern of sleep after each feed to assess the adequacy of milk intake?		
Total score =			

 Occurrence of breast engorgement

Breast engorgement will be assessed through a telephonic conversation, without using any tool. If breast engorgement is present, it will be marked as YES. If breast engorgement is absent, it will be marked as NO.
